# Advanced Electroanatomic Mapping: Current and Emerging Approaches

**DOI:** 10.1007/s11936-024-01034-6

**Published:** 2024-02-27

**Authors:** Sanjiv M. Narayan, Roy M. John

**Affiliations:** 1 Cardiovascular Division, Stanford University, Stanford, CA, USA; 2 Cardiovascular Institute, Stanford University, Stanford, CA, USA; 3 Institute for Computational and Mathematical Engineering, Stanford University, Stanford, CA, USA

**Keywords:** Arrhythmias, Electrophysiology, Ablation, Mapping, Computerized

## Abstract

**Purpose of review:**

Contemporary ablation for complex arrhythmias raises several challenges for electroanatomic mapping. We examine how current and emerging systems may meet these challenges, and we outline major unmet needs.

**Recent findings:**

The latest versions of the 3 major systems (Carto^™^, Ensite X^™^, and Rhythmia^™^) all construct near real-time maps of cardiac anatomy, which can be registered to tomographic images that integrate thousands of electrical points from high-resolution catheters to create activation maps, and display voltage, proprietary features, and ablation lesion locations. While these systems have improved procedural efficiency, it is less clear that they have improved ablation outcomes particularly for atrial fibrillation (AF), scar-related atrial flutter, ventricular tachycardia (VT), and fibrillation (VF). These remain urgent needs.

**Summary:**

Cardiac electroanatomic mapping has reached a mature stage for supraventricular and many ventricular arrhythmias. Novel functional mapping systems have shown success in improving ablation outcomes for AF, VT, and VF in small patient series, but none in randomized trials of broad unselected patient cohorts.

## Introduction

“A map does not just chart, it unlocks and formulates meaning; it forms bridges between here and there, between disparate ideas that we did not know were previously connected.” —Reif Larsen, The Selected Works of T.S. Spivet

In the present-day practice of catheter-based ablations to control cardiac arrhythmias, identification of mechanisms, arrhythmia substrates, and arrhythmia propagation patterns is critical to success. The early days of mapping used a bipolar catheter navigated within heart chambers using fluoroscopic landmarks; the process was imprecise and entailed high X-ray exposure. The advent of three-dimensional electro-anatomic mapping systems (EAM) allowed the creation of an anatomic shell using volumetric data on which to superimpose electrophysiological information relative to this anatomy. Contemporary EAMs have markedly reduced the use of fluoroscopy, and created reliable virtual anatomy for complex mapping and ablation. Other imaging modalities such as echocardiography, CT images, and magnetic resonance images can be incorporated to display areas of scar or other relevant anatomy on an EAM.

Contact-based EAM remains the most commonly used modality, but non-contact systems that incorporate multi-electrode spheroidal [1] or basket [2] catheters have the potential for high-density beat-to-beat mapping when arrhythmias are infrequent. Non-invasive electrocardiographic imaging (ECGI) uses high-density body surface potentials projected on images of cardiac chambers from CT or MRI (ECGI) to generate isochrones and epicardial exits for arrhythmias [3•]. In combination with a non-invasive ablation energy such as stereotactic radiotherapy [4•], such approaches promise truly non-invasive potential future mapping and ablation strategies.

Of the three EAM systems that are currently available to the electrophysiology community (Table 1), familiarity, availability, and local technical support determine the choice of one over the other. It is critical to recognize that all of these systems have limitations and cannot substitute for good understanding of arrhythmia mechanisms, recording, and pacing techniques. This review will bring the reader “up to date” with mapping in the context of current challenges in mapping complex arrhythmias. We synthesize current technologies and techniques, with their best use cases and limitations, then discuss our view of optimal future use cases and technologies.

### Comparison of current technologies

The three most commonly used EAMs are Carto 3 (Biosense-Webster, Johnson and Johnson, Inc.), Ensite Precision (Abbott, Inc.), and the Rhythmia Mapping system (Boston Scientific, Inc., Marlborough, MA).

The Carto system provides magnetic-field based navigation, in which low-level magnetic fields emitted by pads under the patient are detected by location sensors embedded in proprietary mapping and ablation catheters (Table 1). The magnetic field strength detected by each sensor is inversely proportional to the square of its distance to the coil, which is calculated and displayed in a 3D geometry of the heart. Carto 3 adds current-based localization, each electrode emitting current at a unique frequency that can be used to adjust magnetically derived data for a reported spatial resolution of < 1 mm [5]. The system provides an ablation index (AI) which indicates lesion quality by combining stability, contact force, time, and power.

The Ensite system was originally an impedance-based localization and tracking technology (Table 1). Orthogonally situated patches on the patient emit small currents at 8 kHz to create a low-power electrical field; intracardiac catheters read impedance with respect to a reference electrode (e.g., body surface patch) to calculate 3D localization. To correct for non-linear impedance from anatomical non-uniformity within the body, precision uses a process called “field scaling.” The newer X systems add magnetic localization to maintain spatial precision in case of unexpected impedance variations due to volume shifts or other factors [5]. Catheters that unlock this feature are labeled “Sensor Enabled.” EnSite Precision provides indices of ablation lesion quality, and the LSI lesion index combines contact force, radiofrequency application duration, and current [6].

The Rhythmia system (Boston Scientific, MA) uses a hybrid magnetic and impedance-based methodology. The system analyzes unipolar and bipolar signals to eliminate far-field signals based on the maximum negative dV/dt of unipolar ECGs or maximum amplitude of bipolar signals, with high reported accuracy [7–9]. For fractionated signals, the temporal activation of neighboring signals is used to inform the assignment of timing [10]. The system works with a proprietary 64-electrode basket catheter (Orion) and third-party catheters with similar accuracy. The system uses the IntellaNav MIFI^™^ catheters that incorporate 3 mini electrodes (0.8 mm) radially distributed 2 mm from the tip of the catheter. The Directsense^™^ feature uses local impedance at the catheter tip from an electric field generated between the mini-electrodes. It is an indirect measure of tissue contact and is used to monitor the impact of radiofrequency ablation in real time [11].

### What is needed for electroanatomic mapping

In the mid-2020s, it is critical to ask the question “What do I need from my electroanatomic mapping system?” While the major systems may improve the efficiency of ablation procedures, a major unmet need is that they have rarely been shown to improve outcomes—particularly for AF, complex atrial arrhythmias, ventricular tachycardia (VT), or fibrillation (VF).

### Anatomical definition

At their core, all EAMs provide an accurate and stable representation of the anatomy of the chamber of interest. Gating for respiratory and cardiac motion further stabilizes catheter localization within chambers. Other important structures such as the His bundle, fascicles, and course of the phrenic nerve can be tagged on the anatomic shell. Merging of echocardiographic and other imaging can localize structures such as the esophagus, aortic and pulmonary leaflets, origins of the coronary arteries, and important arrhythmogenic structures such as the papillary muscles and the moderator band. Some systems (Table 1) include modules that can superimpose the coronary arteries to improve safety of epicardial ablation.

### Voltage characteristics of the chamber in scar-mediated arrhythmias

A reliable voltage map displaying peak-to-peak electrogram amplitude is cardinal to defining traditional anatomic concepts of substrate—low-voltage regions representing structural abnormalities of fibrosis or scar. Multipolar catheters have greatly facilitated rapid acquisition of large number of points. However, inadequate catheter contact and catheter-induced ectopy remain impediments. Although parameters have been defined for normal and abnormal voltages in the various chambers, it should be recognized that scar tissue can be easily obscured by thin layers of normal endocardium. For example, using a 3.5-mm-tip electrode to a 1-mm ring electrode, a bipolar electrogram amplitude > 1.5 mV is observed in > 95% of sites in the normal ventricular endocardium [12••]. Amplitude < 1.5 mV is associated with fibrosis occupying > 75% of adjacent LV wall thickness. However, normal endocardium overlying fibrosis can generate a > 1.5 mV amplitude signal especially in the non-ischemic cardiomyopathies. Thus, while the < 1.5 mV threshold is relatively specific for fibrosis, its sensitivity is limited. In addition, use of smaller electrodes increases the amplitude of local electrograms relative to remote low-voltage regions [13•]. The ability to alter voltage scale ranges and display of unipolar voltages for a “far-field” view can be helpful in detection of intramural substrates [14].

### Activation versus substrate mapping

Arrhythmia mapping relies on defining activation during an ongoing tachycardia or, if a tachycardia is non-inducible or hemodynamically unstable, definition of its substrate using features that may be implicated in its maintenance. Most scar-related ventricular tachycardia (VT) are mapped and ablated using this latter strategy as 80% of induced VTs can be hemodynamically unstable.

Activation mapping can point to an arrhythmia mechanism. Focal or micro-rentrant tachycardias usually have a centrifugal spread of activation from their exit. Macro-rentrant tachycardias demonstrate continuous activation. Detection requires a window of interest typically equal to the tachycardia cycle length and a clearly identifiable point on the QRS or an intracardiac electrogram selected as the fiducial for timing reference. For focal arrhythmias, it is desirable to have the beginning of the window of interest start shortly before the anticipated time of activation from the focus, such that the rest of the chamber is later. With macroreentry circuits, recordings should encompass a region where activation coded earliest meets the activation that is coded the latest and represent the majority (> 85–90%) of the circuit. Inability to define a complete reentry circuit may indicate incorrect annotation of electrograms, due to limited spatial sampling in the chamber or that part of the reentry circuit is not accessible (e.g., intramural, epicardial, or in an adjacent chamber), or may in fact challenge the mechanism.

Substrate mapping for arrhythmias relies on the detection of potentially arrhythmogenic features. It is important to note that few features are pathognomic for an arrhythmia. Features for VT include low-amplitude fractionated electrograms within scar regions indicating viable conducting channels, late potentials due to delayed conduction, and demonstration of tissue decoupling with pacing (decrement-evoked potentials (DEEP) [15]) or late abnormal ventricular activity (LAVA) [8] (see below). Delayed conduction can be defined by pacing from suspected sites and demonstrating long stimulus to QRS delays (> 40 ms). If the paced QRS approximates VT morphology, its affords further support for the site of pacing in close proximity to a critical isthmus. Potential features for supraventricular arrhythmias and AF include fractionated signals, low voltage representing fibrosis or scar, isochronal crowding suggesting slow conduction at a presumed critical region, and others.

### EAM to assess therapeutic effect of ablation

Current EAMs import precise localization of ablation lesions. With the aid of automated lesion tagging modules based on catheter stability and other parameters, lesion sizing and inter-lesion distances can be used to assess gaps in ablations that aim to create a line of conduction block. The advent of contact force sensing has allowed creation of ablation indices (AIs) that incorporate time, power, and contact force into a single index. In addition, ablation tags can be customized and color coded to include catheter stability, and impedance drops. Proprietary ablation indices (lesion index (LSI) for Ensite Precision and force time integral (FTI) for Carto) correlate with histological lesion formation [16] and can be used to guide ablation for AF [17]. Their utility in ventricular tissue is less well defined. In an ovine model, AI appeared to correlate better with volume than lesion depth [18]. In this study, AI did not correlate with lesion size when half normal saline rather than normal saline was used for open irrigation. Gasperetti et al. used an AI value of 490 for the RVOT free wall and 610 for the RVOT septum with good results and no complications [19].

While ablation indices are useful as a rough guide to lesion creation, lesion durability is less well defined due to wide variability in wall thickness [20], tissue remodeling, disease progression, and other factors. Several considerations, such as the lowest AI that would prevent collateral damage to the esophagus, are undefined. Alternative energy forms such as pulse field ablation with a high threshold for non-cardiac tissue provide one likely solution [21].

### Innovations in electrical recording technologies

**Fig. 1:**
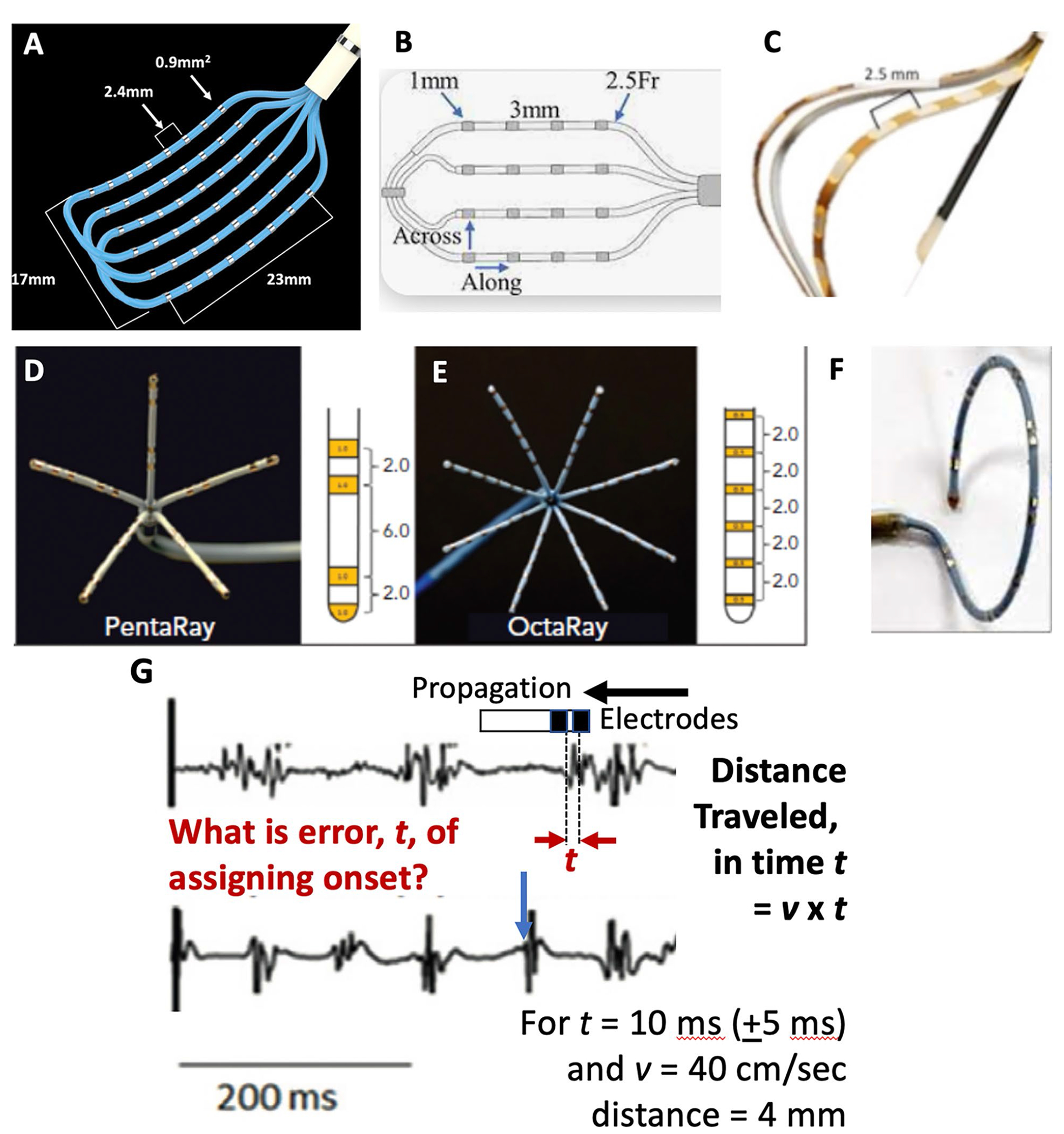
Multipolar catheter designs and resolution: **A** Optrell^™^, **B** HDAdvisor Grid^™^, **C** Orion^™^, **D** Pentaray^™^, **E** Octaray^™^, and **F** Lasso^™^, each with ~ 1–2-mm electrodes spaced ~ 2–3 mm. **G** Required spatial resolution can be determined by the distance traveled by the arrhythmia wavefront within the timing error. For precision ± 5 ms and conduction velocity 40 cm/s (low–normal), 4 mm spacing is sufficient. Slower conduction and less error (e.g., simple, *blue*, versus complex, *red*, electrograms) will require higher resolution. **A**, **D**, **E**, **F** Permissions from Biosense-Webster, Inc. **B** Permissions from Abbott Laboratories. Advisor is a trademark of Abbott or its related companies. Reproduced with permission of Abbott, © 2023. All rights reserved; **C** Used with permission of Elsiever, from [7]; permission conveyed through Copyright Clearance Center, Inc.).

EAM continues to advance, in part, by incorporating recordings at ever higher spatial density, facilitated by several high density multipolar catheters. The spatial configuration of some catheters shown in Fig. 1 is tailored to specific applications, such as mapping the circular orifices of pulmonary veins (Lasso^™^), or vectors of activation (HDGrid^™^, Optrell^™^). Newer electrode designs are being tested, such as circular en face concentric electrodes for certain applications [22].

Defining the spatial resolution required for mapping is an important issue. While higher spatial resolutions are intuitively superior, optimal resolution cannot be separated from the precision of measurement and arrhythmia physiology. For instance, if the precision of marking activation time (error) is ± 2.5 ms (i.e., a 5-ms range), an activation wave at a normal conduction velocity of 40–100 cm/s will cover (40 × 0.005 to 100 × 0.005) 0.2 to 0.5 cm in this time. Thus, 2–5 mm may represent a relevant resolution to map isochrones in atrial or ventricular arrhythmias in normal hearts. If marking precision is lower, however, such as for complex AF electrograms with precision of ± 5 ms (10-ms range) [23•] or worse, a resolution of 4–10 mm may suffice for the same conduction velocity, with smaller electrode spacing if conduction is very slow. Note that even with automated marking [8], precision beyond ~± 2.5 ms may not be achievable due to biological variability. The optimal electrode size is unclear. While electrodes summate electrical field strengths according to the inverse square law, this has yet to be translated into a recording distance (or sensing antenna dimension). Takigawa et al. recently confirmed intuition that smaller electrodes better identify near-field and remove far-field signals [13•].

There is continued interest in unipolar recordings. Historically, unipolar maps were used to precisely identify local activation from the maximum negative dV/dt. However, far-field signals captured between the electrode tip and remote indifferent patch caused a preference towards bipolar recordings that subtract similar (“common”) far-field between electrodes of the bipole. Nevertheless, unipolar electrograms retain potentially useful electrogram shape information, and in AF avoid artifacts introduced by subtracting signals from different potential wavefronts. Unipolar signals on the endocardium may also be used to detect epicardial and intramural scar [14]. This has been used to “reconstruct” 3D activation by simultaneous endocardial and epicardial mapping from the vasculature and from intramural penetrating vessels [24].

### Newer techniques in arrhythmia mapping

The availability of high-density EAM has triggered a number of investigations into improved mapping and safe ablation of common supraventricular arrhythmias, especially in the pediatric population. Recent studies also emphasize the incorporation of arrhythmogenic substrate imaging to allow substrate modification for scar-related ventricular tachycardia.

### Mapping techniques for supraventricular arrhythmia

#### Slow pathway localization

**Fig. 2:**
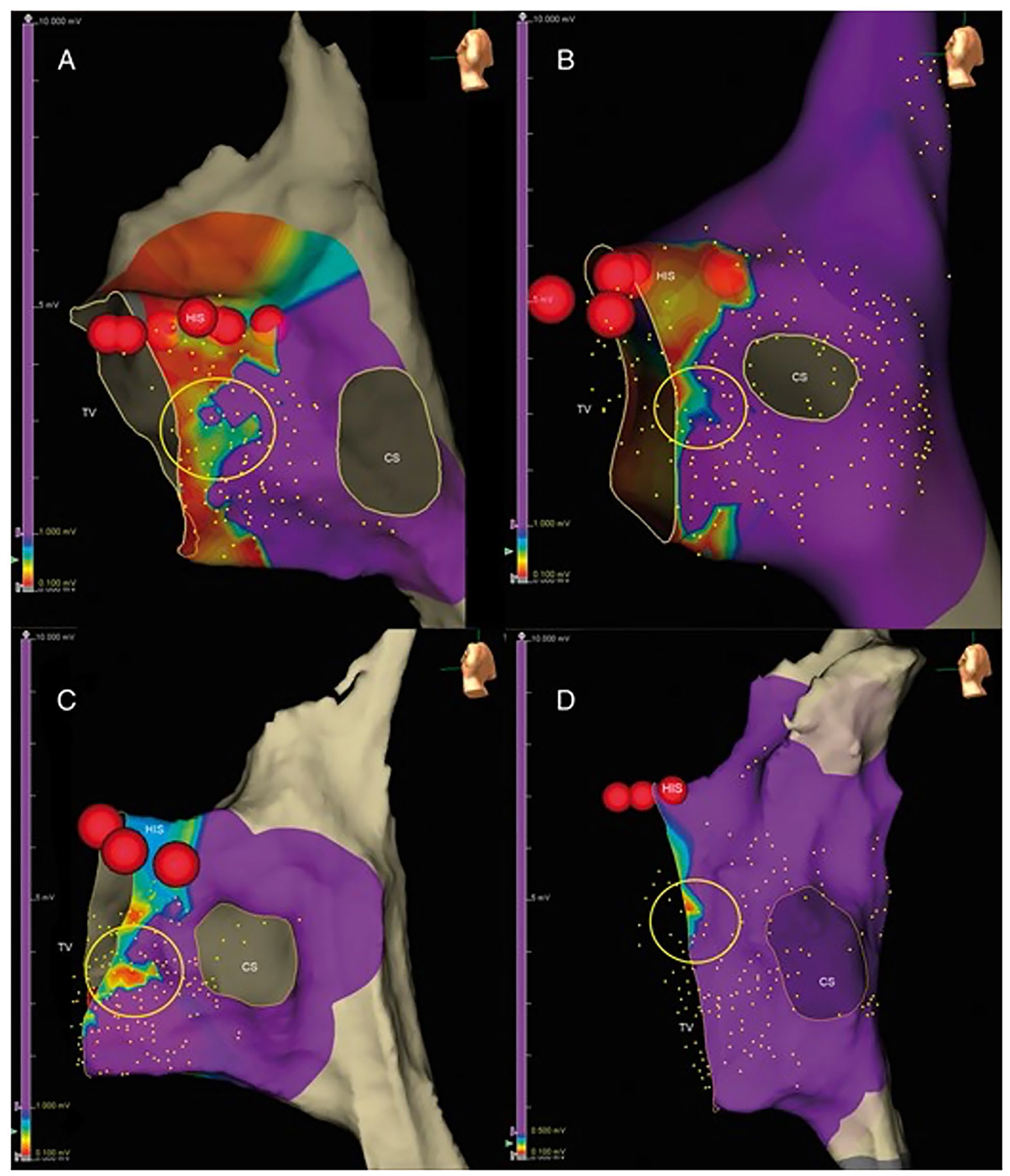
Low-voltage bridge identified using electroanatomic mapping in AVNRT: **A**, **C** type I and **B**, **D** type II (highlighted with a *yellow circle*). The *red dots* are the site of His-bundle recording. CS, coronary sinus; His, His bundle (*red dots*); TV, tricuspid valve. (Used with permission of Oxford University Press, from [25]; permission conveyed through Copyright Clearance Center, Inc.).

High-density mapping of the triangle of Koch has sought to further define the anatomy and physiology of the slow pathway area to facilitate ablation for AV nodal re-entrant tachycardia. Single-center studies describe a low-voltage bridge (LVB) that can be discerned in a field of high voltage between base (coronary sinus ostium) and apex of the Koch’s triangle. Two patterns may be observed (Fig. 2). Ablation at the site of the bridge has a high chance of suppression of slow pathway function. However, the successful site may still be 2–4 mm away from the point of the LVB in 50% of patients [25]. Some investigators have described the LVB as a non-specific finding present in patients without AV nodal re-entry.

Other novel tools have been incorporated into electroanatomic mapping to assist in ablation of AVNRT and other arrhythmias. Ripple mapping displays peak-to-peak voltages, rather than activation times, as a movie of 3D bars changing in length as a function of their voltage–time relationships emerging from the anatomical surface. In Koch’s triangle, such ripples [26] can reveal prolonged, low-amplitude signals which helped identify sites for cryoablation in a pediatric population with fewer lesions than conventional mapping.

### Open window mapping for accessory pathway-mediated SVT

**Fig. 3:**
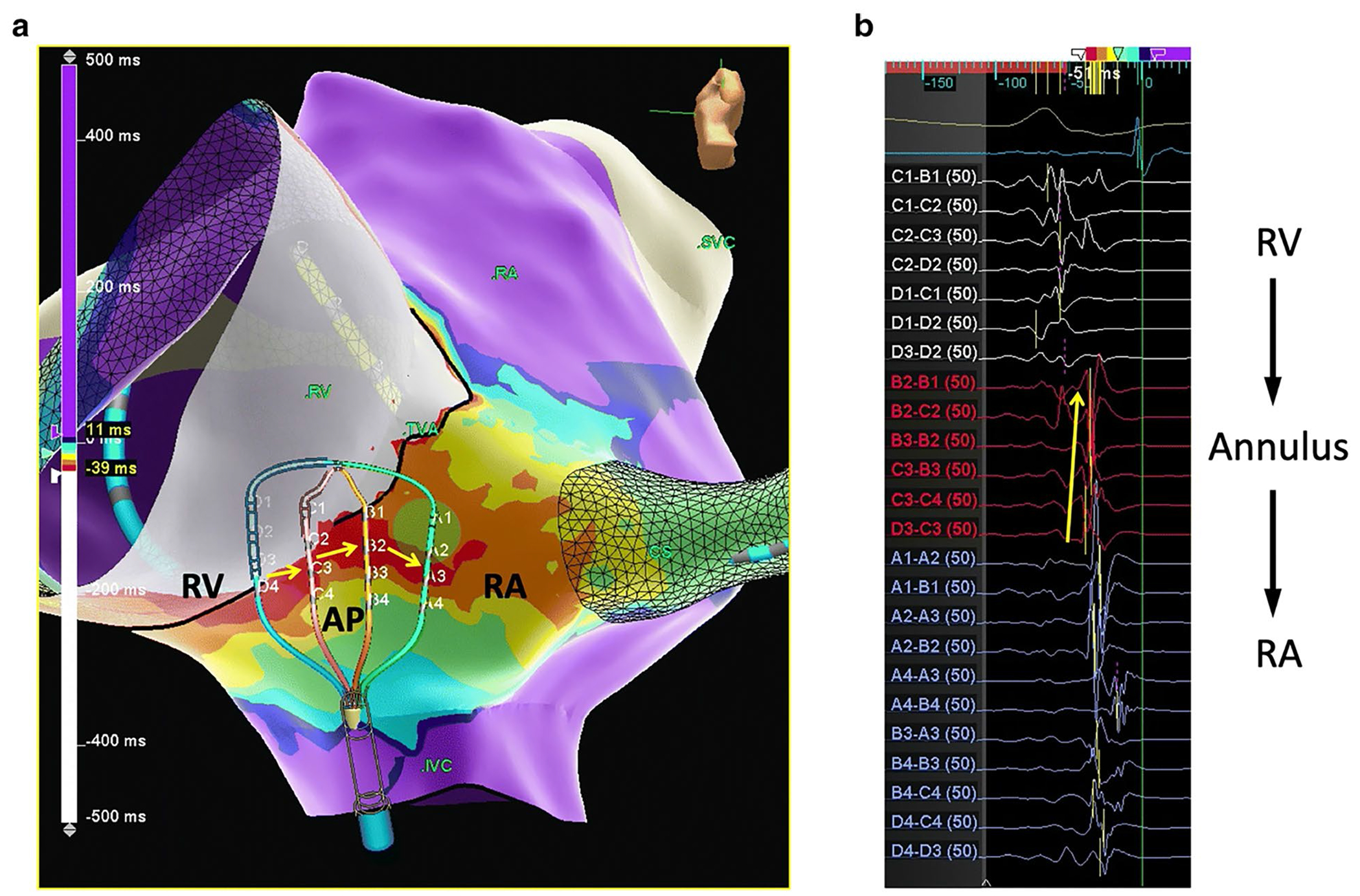
Open-window mapping of a right posteroseptal accessory pathway causing orthodromic reentrant tachycardia. **a** Multi-electrode mapping catheter spanning the accessory pathway. *Yellow arrows* indicate direction of activation from the RV across the accessory pathway to the RA. **b** Electrograms at this site with annotations (*thin vertical bars*) calculated automatically here at > 800 points in 8.1 min. AP, accessory pathway; RA, right atrium; RV, right ventricle. *Yellow arrow* denotes activation sequence across the tricuspid annulus. (Used with permission of Springer, from [27]; permission conveyed through Copyright Clearance Center, Inc.).

Accessory pathway localization can be difficult when contact of a ablation catheter along the AV annulus is unstable. High-density multipolar electro-anatomic mapping can facilitate detection of gaps in conduction block across AV valves (i.e., accessory pathways) using a technique called “open window mapping” (Fig. 3). The technique is based on an automated annotation processs in which signals with the highest – dV/dt are automatically annotated at each point irrespective of the cardiac chamber. The accessory connection is visualized as a breakout of activation into the chamber of interest (atrium for VA conduction or ventricle during AV conduction). In a study using the HD grid catheter in the Ensite electroanatomic mapping system (Abbott Inc.), Schricker et al. utilized “open window” mapping to detect both atrial and ventricular activation [27]. Earliest site of breakout in the chamber of interest was detected as the earliest maximum dV/dt. In this single-center study, ablation was successful in 100% of 23 patients with a mean of 18.5 s of ablation. Open window mapping has also been performed using the Octaray catheter in the Carto electroanatomic mapping system [28]. Whether this technique adds considerably to conventional catheter-based mapping techniques remains to be seen.

### Recent progress in EAM utilization for mapping and ablation for VT

**Fig. 4:**
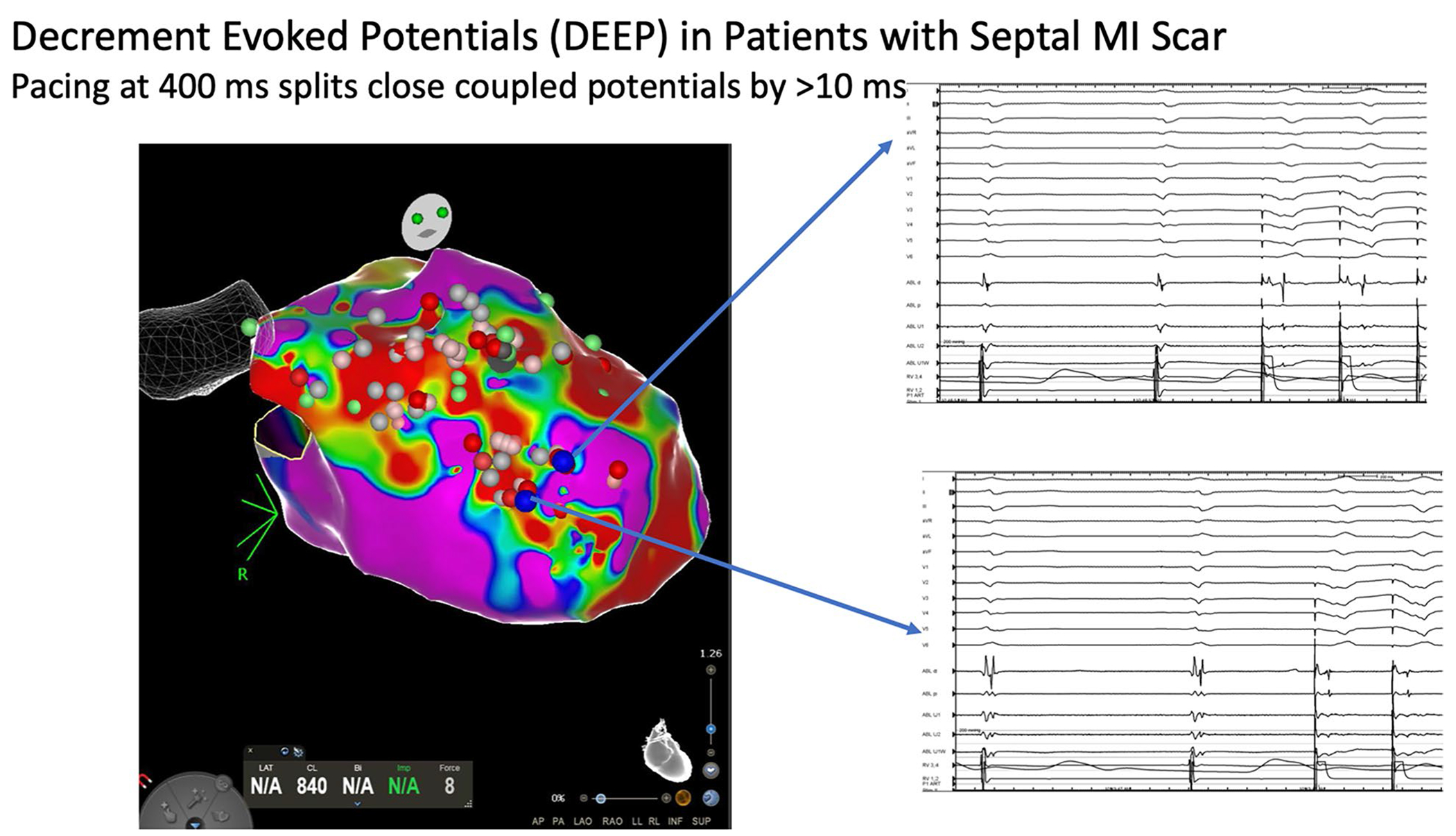
Decrement-evoked potential (DEEP) mapping in a patient with an anterior septal scar and VT. Pacing at 400 ms at sites indicated by *blue tags* separated high-voltage electrograms from far-field signal (decrement-evoked potentials), which can be targeted for ablation. *Blue arrows* point to electrogram recordings from the left ventricular endocardial areas indicated by the *blue dots* on the electroanatomic map.

In substrate mapping for VT, the EAM has been used to annotate emerging targets. Late potentials are ubiquitous in large infarct scars, and thus have limited positive predictive value for identifying critical isthmuses. Local abnormal ventricular activation (LAVA) represented by low-amplitude high-frequency local electrogram occurring during or after far-field electrogram has a higher predictive value [29]. More recently, the use of extrastimului or rapid pacing has been used to separate out high-frequency signals in a technique called decrement-evoked potentials (DEEP) [15] (Fig. 4). The greater the decrement, the higher the specificity for a VT substrate. In one study, DEEP-based approach for ischemic cardiomyopathy rendered VT acutely non-inducible in 80% of patients [30].

**Fig. 5:**
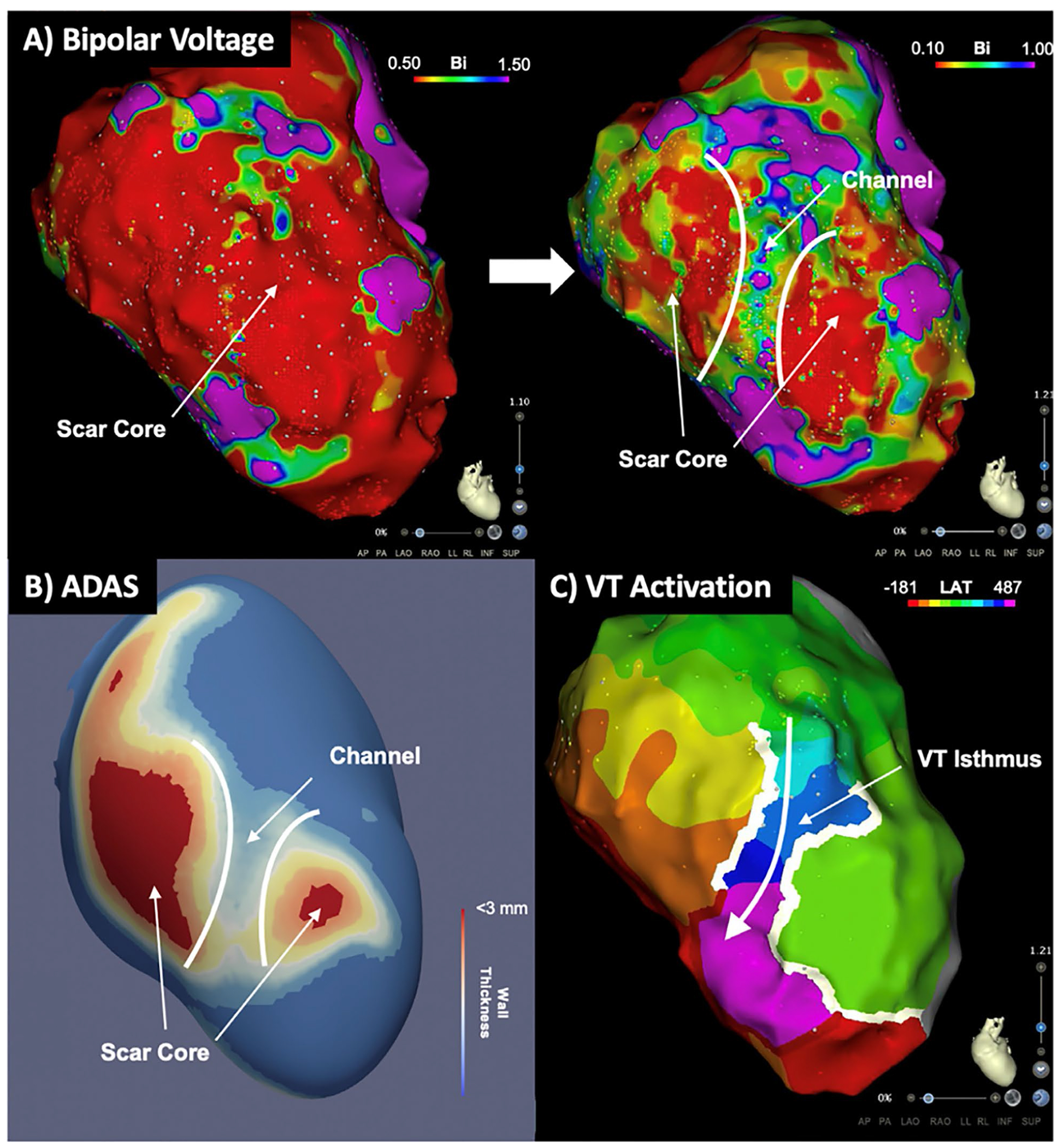
Voltage mapping with and without ADAS in a 72-year-old male with previous anteroseptal infarct: **A** voltage mapping using traditional range of 0.5–1.5 mV shows a large area of scar core over the antero-septum with no definite channel. Adjusting the voltage window to 0.1–1.0 mV reveals a channel between two areas of dense scar. **B** Pre-procedural cardiac CT with automated detection of arrhythmogenic substrate (ADAS). **C** Activation map of clinical VT, demonstrating conduction through the conducting channel identified with both ADAS and voltage mapping. (Used with permission from Dr. Geoffrey Lee, from [31]).

Incorporating preprocedural CT or MRI to an EAM can further assist with identification of channels. One approach that implements automated detection of arrhythmogenic substrates (ADASs) defines areas of dense scars with border zone corridors that may act as channels for VT. Preliminary studies using this software are promising [31], but prospective validation is awaited. Note that simple manipulaton of voltage windows on a traditional EAM voltage map alone can display channels that can correspond to ADAS acquired data (Fig. 5).

Isochronal late activation mapping (ILAM) is a form of functional substrate mapping to identify slow conduction properties of potential arrhythmia substrates. Automated ILAM is performed in EAM using a software module that annotates local timing to the last deflection of the local electrogram (EnSite Precision, Abbott Park, IL) to identify isochronal crowding that may relate to critical sites for ablation of VT [32].

### Novel approaches to map wavefront direction

Propagation perpendicular to a bipole generates zero amplitude, and thus can result in > 50% variations in electrogram amplitude compared to parallel wavefronts. Vectorial approaches have been applied to calculate propagation, which may help map arrhythmia sources. Omnipolar mapping using catheters with electrodes arranged in a spatial grid, such as the HD Grid^™^ (Abbott) and Optrell^™^ (Biosense), is a novel solution to calculate multiple bipole directions that minimize loss of electrode amplitude. Such vectorial approaches can better detect low voltage [33], which is otherwise sensitive to wavefront direction [34]. Recent studies have questioned the accuracy of this approach for vectorial wavefront analysis, and have suggested other vectorial approaches that should be tested in clinical studies [35]. Alternative approaches by the Carto system estimate ambiguous activation times from neighboring sites [36].

### Emerging mapping systems

Several mapping systems are being tested as an adjunct to mainstream EAM systems. Their main motivation is to provide physiological information for specific arrhythmias that may not be available from traditional EAM data inputs or analyses.

One example to guide VT ablation is the InHeart^™^ system to map potentially arrhythmogenic wall thinning from ventricular CT scans using computational digital twin models [37]. In addition, CT imaging with InHeart segmentation has been used to detect lipomatous metaplasia or infiltrative fat (inFAT) in chronic infarcts scars [38, 39]. Fatty infiltration is a late manifestation of infarct remodeling and tends to occur in close proximity to substrates for VT and is associated with higher incidence of VT. EAM in areas of inFAT shows low voltage and prolonged electrogram duration. The presence of inFAT by CT imaging was associated with a worse mortality and VT free survival [38]. How it influences the current practice of VT ablation is unclear and needs further investigation.

Several systems are under development to map atrial fibrillation (AF). Traditional AF mapping aims to confirm pulmonary vein isolation (PVI) and identify gaps in prior lines [1]. Nevertheless, the success of PVI remains ~ 50–75% over 12–18 months [40, 41, 42•] despite advances in mapping and in the durability of ablation lesions. This has motivated continued efforts to advance mapping to identify ablation targets outside the PVs, which are particularly relevant for patients with AF and isolated pulmonary veins after prior ablation (> 60% of repeat ablations in contemporary practice [43, 44]). merging approaches are still in development, but attempt to identify specific electrogram features [45•, 46, 47], repetitive, focal, or rotational activity [48–50, 51•, 52] involved in AF maintenance.

**Fig. 6:**
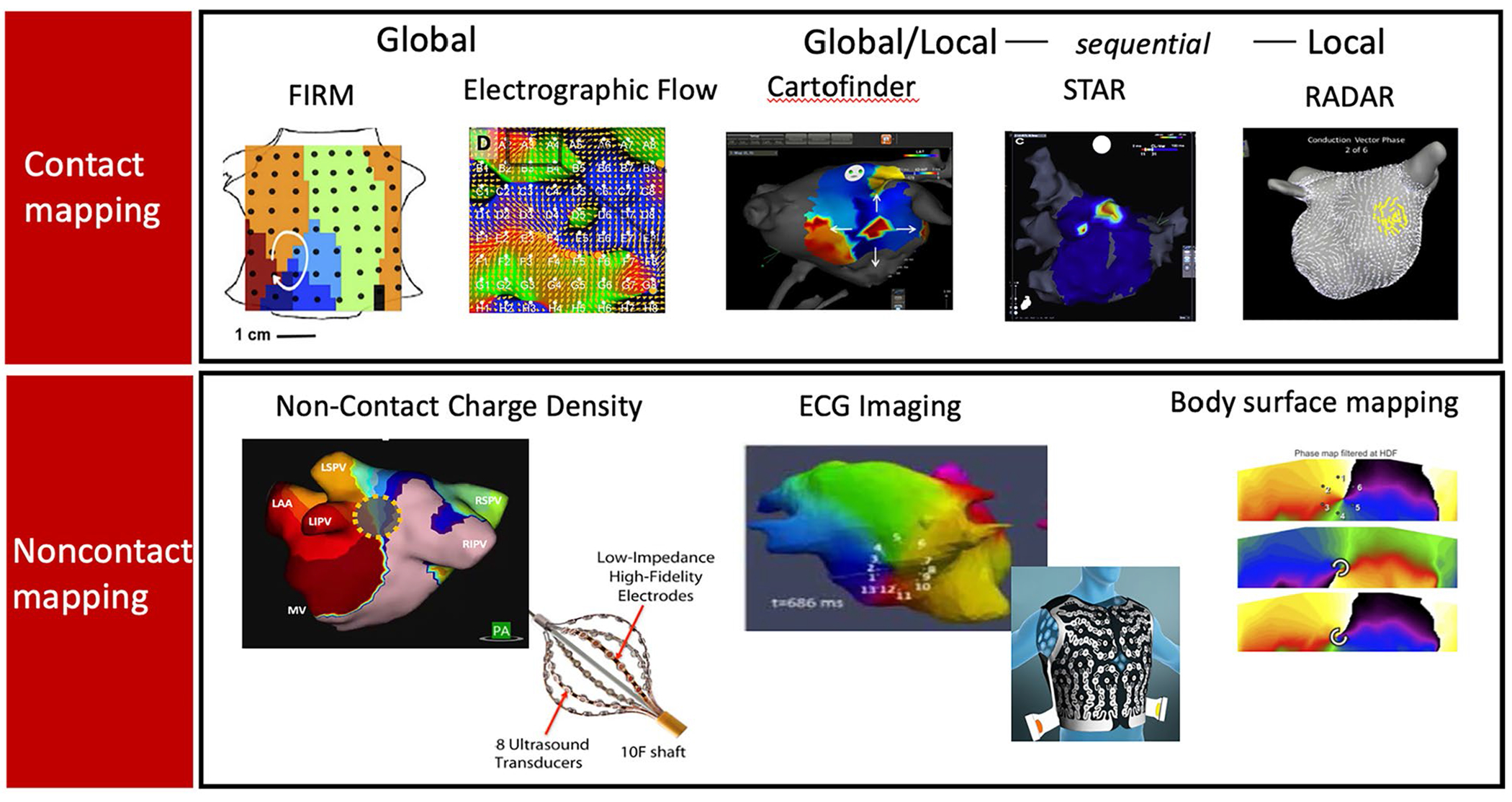
Emerging mapping systems for atrial fibrillation.

Table 2 and Fig. 6 summarize existing and emerging AF clinical mapping systems. An early AF mapping system (Rhythmview^™^, Abbott, IL) identified localized sites of focal and rotational activity, which was shown to provide ~ 80% concordance with simultaneous optical mapping of human AF [24]. Subsequent systems have been developed to improve algorithmic specificity for such sites using similar basket catheters [53] or non-contact mapping [54], or to identify electrical signatures of such pathophysiology [55].

However, such systems are controversial. While several have shown success in small single or multicenter studies, none have improved AF ablation success in randomized multicenter studies. Notably, these systems demonstrate substantial heterogeneity in success between centers [48–50, 51•], which may need to be resolved before larger trials can be successful.

Main areas for additional research and development include recording approach, how to target such sites for ablation, and ablation endpoints. Panoramic contact basket catheters enable global recordings of AF, particularly in recent designs that improve atrial coverage over prior designs [56], yet often provide suboptimal contact and modest resolution. High-density contact mapping fits well with current workflows, but mandates sequential recordings that introduce challenges of registering these regions in a temporally fluctuating arrhythmia such as AF. On the other hand, it is possible that spatially consistent regions may be resolved by this approach, which is the basis for several systems in Table 2.

## Conclusions and future directions

Electroanatomic mapping has revolutionized our understanding and treatment of cardiac arrhythmias in the past two decades. EAM is now a central pillar in interventional therapy for a wide array of supraventricular and ventricular arrhythmias, and has been shown to improve safety, efficiency, and, in some arrhythmias, efficacy. Current EAM systems are comprehensive and accurate. Nevertheless, EAM systems need to further evolve and improve to meet the challenges of complex ablation, particularly to improve outcomes for the ablation of AF and VT in patients with structural heart disease. Bioengineering innovations are poised to meet these challenges in coming years, but will need to be combined with major pathophysiological insights and improved design of ablation endpoints for clinical trials. We eagerly anticipate continued development in this area.

## Figures and Tables

**Table 1.** Features of the mapping systems in common use

	Abbott NavX	Biosense-Webster	Boston Scientific
Trade name	Ensite Precision (older) X (new version)	Carto 3 (new) Several preceding versions	Rhythmia
Principle of localization	Electrical impedance (Precision and earlier); and magnetic (Ensite X)	Magnetic field	Hybrid magnetic and impedance based.
Principal advantages	Flexible system, open platform to desired catheters Ensite X: localization relies on stable external patch Elimination of artifact from respiration/motion	Localization accuracy, assisted by closed system (proprietary only catheters) Localization relies on stable external patch	Flexible system: can use Proprietary (Orion) and other catheters. Claimed accuracy of signal tagging, high fidelity for small high frequency signals.
Disadvantages	Limited utility in non-sustained or varying arrhythmias Precision: localization relies on reference catheter stability; may need remap if this moves	Limited utility in non-sustained or varying arrhythmias Incompatibility with non-proprietary catheters Limited display of diagnostic reference catheters	Limited utility in non-sustained or varying arrhythmias
Localization hardware	Precision: electrical current at 8 kHz from orthogonal skin patches, catheter location identified by impedance at tip X: magnetic field emitted by patches, sensed by catheter	3 pads under table, emit low-magnetic field (5 × 10^−6^ to 5 × 10^−5^ T). Six patches on chest. Sensed by catheter, converted to distance from coil	
Catheter	Precision: open system—any catheter sensed by impedance (with calibration) Ensite X—magnetic field sensed by proprietary catheter	Proprietary NavStar catheter (with sensor) detect field Carto 3 adds current localization: catheter emits current at a unique frequency	Compatible with several catheters; 200 intracardiac channels Proprietary Intellamap^™^ mapping catheter. (8F deflectable mini-basket of 1.8-cm diameter, 8 splines 8 electrodes 2.5-mm spacing) and IntellaNav^™^ ablation catheters
Localization accuracy	Reported < 1–2 mm	Reported < 1 mm [5]	Reported < 2 mm [57], 0.01 mV noise floor
Ablation tagging	Tags ablation points by parameters of contact force, impedance drop, stability and power	Tags ablation points by parameters of contact force, impedance drop, stability and power	Tags ablation points by several parameters including impedance drop, stability, and power from IntellaNav catheter.
Multimodality image integration	Verisimo: segments CT and MR images Ensite Fusion Registration module ADAS to integrate CT, MRI	Segments CT and MR images CartoSound: Incorporates echocardiographic images to the EAM Superimposes coronary angiography images on EAM	Multiple
Other mapping modules	Omnipolar mapping from HDGrid^™^ catheter to improve activation mapping by less directional assessment Ensite OT module for accurate electrogram annotation LiveView movies created on HDGrid catheter in near real time Connected Care modules for device connectivity	Cartofinder: finds repetitive and rotational activity Ripple mapping of bipolar voltage to identify activation waves and low voltage Voltage mapping with proximity markers Confidense^™^, Coherence^™^. automated unipolar and bipolar wave-front annotation [58] Pattern matching to compare spontaneous or paced QRS pattern with arrhythmia	Improves accuracy of mapping [7–9] by using unipolar and bipolar signals to eliminate far-field signals based on the maximum negative dV/dt of unipolar ECGs or maximum amplitude of bipolar signals.

**Table 2.** Systems focused on mapping atrial fibrillation to guide ablation

	Mapping system	Basis for mapping	No. targets	Atrial location	Target characterization	Freedom from AF at 12 months
**Contact mapping with Baskets**	**(RhythmView^™^)** [10, 25, 26]	Activation or phase mapping; signals filtered by published rate response of human atrial action potentials and conduction velocity [59–62].	3–5	LA 70% RA 30% PV 24%	Stable rotations 76% Focal sources 24% [10]	Paroxysmal and non-paroxysmal AF. Meta-analysis: 72.5% [7] Persistent AF RCT: 77.7% (FIRM + PVI subgroup) [27]
	**(Ablacon^™^)**	Electrographic flow mapping [28, 29]	4–6	LA 70% RA 30% PV 40%	Rotational 51% Focal 49%	Pending
**Sequential contact mapping**	**CartoFinder^™^ (Biosense Webster)** [30–32]	Early activation based on qS shape on unipolar electrograms, rotational activation	1–3	LA 63% RA 27% Non-PV 79% [33]	Rotational activity 70% Focal activations 30% [30] to 100% [32]	Persistent and long standing persistent AF 71% [32] 70% [33]
	**Spatiotemporal dispersion (Volta Medical)** [63•]	Dispersion of electrograms identified by AI and models of rotational activity	12	LA 80% RA 20%	Electrogram dispersion, representing micro-reentry	Persistent AF 86% success with or without drugs (1.3 procedures, 12 months)
	**Stochastic Trajectory Analysis of Ranked Signals (STAR^™^)** [35]	Sites of consistently early activation in AF, ranked stochastically	2–3 (post PVI)	LA 95% RA 5%	Early sites of activation	Persistent AF 80% (AT/AF at 18 months)
	**Real-time electrogram analysis for drivers of AF** [36] **(CardioNXT^™^)**	Vectorial analysis of activations, spatially aggregated based on similar coronary sinus patterns	3.9 ± 1.3 (LA) 2.5 ± 1.4 (RA)	Inconsistent RA mapping	Rotational (73%) and focal sites	Persistent AF Long-standing AF 74% AF freedom at 13 months (on/off drugs)
**Non-contact mapping**	**Charge/dipole density** [37, 38] **(Acutus^™^)**	Dipolar, ionic activation	2–3	RA not mapped LA anterior 70%	Localized irregular activity Localized rotational activity Focal activity	Persistent AF 73% [38]
	**Body surface, ECGI** [13•, 39, 40] **(CardioInsight^™^, EP Solutions^™^)**	Inverse solution to infer epicardial electrograms. Activation and phase mapping.	3–6	LA 70% RA 30% LPV/LAA 82% [13•] LA 53% RA 27% Septum 20% [39]	Re-entries 80% Focal breakthrough 20% [13•]	Persistent and long standing persistent AF 85% [13•] 78% [39]

## Opinion statement

Choosing between established mapping systems largely reflects operator preference. However, such systems have not convincingly improved outcomes for ablation of AF, VT, and VF. Ultimate solutions to this “last frontier” must address the clear variability in arrhythmia phenotypes between patients, and translate these insights to efficient and robust procedures that improve outcomes. We eagerly anticipate novel approaches to this space.
